# α-Smooth Muscle Actin and ACTA2 Gene Expressions in Vasculopathies

**DOI:** 10.5935/1678-9741.20150081

**Published:** 2015

**Authors:** Shi-Min Yuan

**Affiliations:** The First Hospital of Putian, Teaching Hospital, Fujian Medical University, Putian, China

**Keywords:** Actins, Aorta, Thoracic, Mutation, Missense

## Abstract

α-smooth muscle actin, encoded by ACTA2 gene, is an isoform of the vascular smooth muscle actins, typically expressed in the vascular smooth muscle cells contributing to vascular motility and contraction. ACTA2 gene mutations cause a diversity of diffuse vasculopathies such as thoracic aortic aneurysms and dissections as well as occlusive vascular diseases, including premature coronary artery disease and ischemic stroke. Dynamics of differentiation-specific α-smooth muscle actin in arterial smooth muscle cells and proliferation of the proteins have been well described. Although a variety of research works have been undertaken in terms of modifications of α-smooth muscle actin and mutations of ACTA2 gene and myosin, the underlying mechanisms towards the pathological processes by way of gene mutations are yet to be clarified. The purpose of the present article is to describe the phenotypes of α-smooth muscle actin and implications of ACTA2 mutations in vasculopathies in order to enhance the understanding of potential mechanisms of aortic and coronary disorders.

**Table:** 

**Abbreviations, acronyms & symbols**	
ACTA2	= Actin, alpha 2, smooth muscle, aorta
bFGF	= Basic fibroblast growth factor
CAD	= Coronary artery disease
HH	= Hamburger - Hamilton stage
MYH11	= Myosin heavy chain 11
MYLK	= Myosin light chain kinase
PDGF	= Platelet derived growth factor
SMA	= a-smooth muscle actin
SMCs	= Smooth muscle cells
TAAD	= Thoracic aortic aneurysm and dissection
TGF	= Transforming growth factor
TGF-β_1_	= Transforming growth factor beta 1
TGFβR1	= Transforming growth factor beta-receptor 1
TGFβR2	= Transforming growth factor beta-receptor 2

## INTRODUCTION

The pathogenesis of vasculopathies, including aortic and coronary disorders, are uncertain, even though they have been under constant investigation. The potential roles that α-smooth muscle actin (SMA) might play in the development of vasculopathies have drawn much attention.

SMA, encoded by ACTA2 gene, is an isoform of vascular SMA. The major functions of vascular smooth muscle cells (SMCs) are typically vascular motility and contraction, and the functions of certain non-muscle cells, such as myofibroblasts, are healing wounds, scars and fibrocontractive lesions^[1]^, depending on the cyclic interaction between thin filaments composed of the SMC-specific isoform of α-SMA encoded by ACTA2 and thick filaments composed of SMC-specific β-myosin. α-SMA and the encoding gene ACTA2 mutations have been frequently described in relation to pathogenesis of vasculopathies in clinical settings^[2]^. Studies on ACTA2 mutations have demonstrated that mutation carriers show various vasculopathies, including premature onset of coronary artery disease (CAD), premature ischemic strokes (including Moyamoya disease), and familial thoracic aortic aneurysms and dissections (TAADs)^[2]^. Proliferative and secretory activities as well as transition from a contractile to a synthetic phenotype of α-SMA were considered the underlying mechanism of vasculogenesis^[3]^. Experimental studies with explanted SMCs and myofibroblasts from patients harboring ACTA2 revealed that increased proliferation of SMCs contributed to occlusive diseases. Although a series of research works have been undertaken in terms of α-SMA modifications and ACTA2 gene mutations, the underlying mechanisms of pathological processes by way of mutations remain underestimated. In order to highlight the potential mechanisms of pertinent vasculopathies and to enhance management strategies, the phenotypes of α-SMA and implications of ACTA2 mutations in vasculopathies are described.

### Molecular structure of α-SMA

There are four distinct variants of actins: two vascular SMC (α-SM and γ-SM)-specific, and two cytoplasmic actins (β-NM and γ-NM actins) in eukaryotic cells. α-SMA is located primarily in the microfilament bundles of vascular SMCs and exerts contractile functions. It shows a close relationship with arterial tones and the activation of myofibroblasts^[4]^. The forceful contractile property of myofibroblasts is attributable to the stress fibers dependant on α-SMA^[5]^. SMC components and extracellular matrix (collagen and elastin) are determinants of the contractility, distensibility and elasticity of the aortic wall. Extracellular matrix disruption or imbalance of the extracellular matrix components leads to stiffness of the aortic wall and further development of TAAD^[6]^. In experimental homozygous α-SMA knock-out mice, deficits of vascular contractility and reduced basal blood pressure were noted, implicating the important role of α-SMA in maintaining vascular tone^[7]^.

### Clinical investigations

Normal aortic media predominantly contains α-SMApositive SMCs with homogeneous elastic laminae^[8]^ whereas degeneration is marked by disruptions of α-SMA-positive SMCs^[9]^. Vascular occlusions are pathologically characterized by medial proliferation of SMCs or enhanced proteoglycan accumulation along with fragmentation and loss of elastic fibers^[10]^. The α-SMA mutations in TAAD hinder the binding affinity between the monomer and the ligands, thereby generating more unpolymerized monomers^[10]^. The aortic media of abdominal aortic aneurysm displays fewer α-SMA-positive cells, which are secondary to extensive aortic wall structural damages. Medial SMC density may decrease by 79.1% in abdominal aortic aneurysmal tissue in comparison with that of normal control^[8]^. Aortic aneurysmal wall shows chronic infiltration of the inflammatory cells, degradation of the extracellular matrix, and apoptosis of SMCs^[11]^. In addition, macrophages express chemoattractant proteins and matrix metalloproteinases in the atherosclerotic plaques^[12]^ similar to aortic aneurysm, in particular in the aneurysm necks for the stent graft anchoring.

An immunohistological study has shown more α-SMA expressions in coronary erosion lesions than in stable plaques or mildly stenotic plaques^[13]^. Acute arterial injury was found to be associated with changes in glycosaminoglycans of the extracellular matrix ahead of those of SMC phenotype, whereby matrix glycosaminoglycans are substituted by SMCs rapidly during the modification process where neointima does not generate enough heparin sulphates^[14]^.

Coronary stenting may cause concentric narrowing of the lumen due to neointimal formation, in which the extracellular matrix increases and number of SMCs decreases over time. The neointima is composed of three-layered and circular-arranged α-SMA-positive cells. Immunohistochemical studies of the common carotid artery with stent deployment showed in-stent thrombus around the stent struts with the neointima composed mainly of α-SMA-positive cells within type I collagen-positive matrix^[15]^. Comparative studies on the abdominal aortopathies revealed that α-SMA-positive SMCs were predominant in the aortic media of the occlusive abdominal aorta and the aneurysmal neck; however, SMCs were disrupted and disarrayed in the aneurysmal body, in which much fewer SMCs were present than in the occlusive aorta and the aneurysmal neck^[16]^.

Transforming growth factor (TGF)-β-antagonist reduces α-SMA expression, and treatment with TGF-β may upregulate α-SMA expression. TGF-β_1_-stimulated adventitial fibroblasts in TAAD patients attenuated extracellular matrix production and SMC transformation^[6]^. In the aneurysmal neck, there was increased TGF-β_1_ expression and reduced SMC density, and even more upregulated TGF-β_1_ expression and much more reduced SMC density were found in the aneurysmal body in comparison with those of the occlusive aorta. Therefore, a link between TGF-β_1_ overexpression and reduced SMC density as a result of SMC apoptosis and attenuated SMC proliferative capability may characterize the aneurysmal formation^[16]^.

### Genetic aspects of ACTA2 mutations

**Fig. 1:**
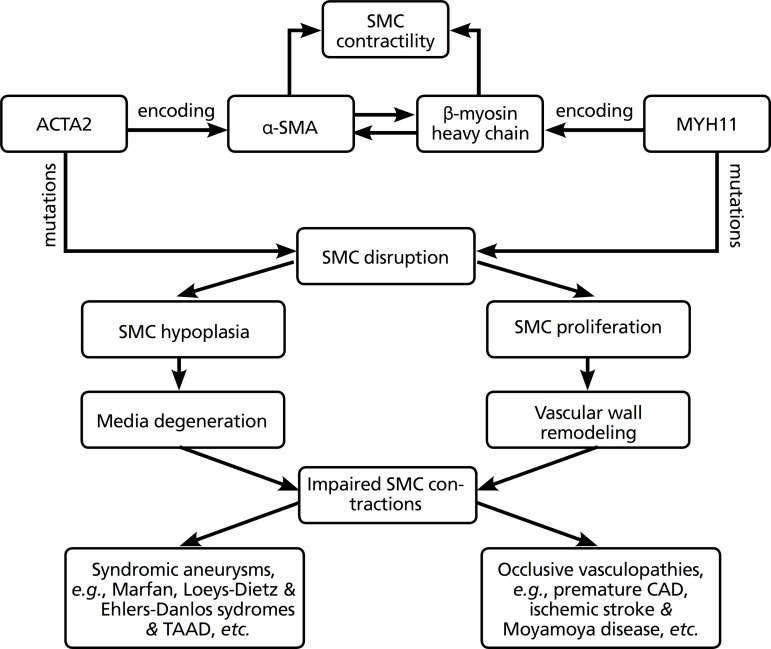
The relationship among smooth muscle actin, ACTA2 gene and contractile property in vasculopathies. The contractility of the smooth muscle cells is maintained via cyclic interactions between α-smooth muscle actin (encoded by ACTA2) and the β-myosin heavy chain (encoded by MYH11). Missense mutations in ACTA2 and myosin are responsible for the development of syndromic aneurysms or occlusive vascular disorders, depending on the vascular pathology of either medial smooth muscle cell hypoplasia or medial proliferation. ACTA2=actin, alpha 2, smooth muscle, aorta; CAD=coronary artery disease; MYH11=myosin heavy chain 11; SMA=smooth muscle actin; SMC=smooth muscle cell; TAAD=thoracic aortic aneurysm and dissection

ACTA2, the encoding gene of α-SMA, located at 10q22-q24 and composed of eight coding exons, is a major contractile component of the arterial SMCs^[2]^. ACTA2 gene defects that cause familial TAAD type 6 have been reported^[17]^. So far, more than 20 different missense mutations of ACTA2 have been found to be associated with aortopathies^[18]^. ACTA2 mutations may show reduced penetrance and variable expressivity due to a dominant negative mechanism^[19]^. Heterozygous ACTA2 mutations may lead to impaired SMCs, showing pathological hyperplasia and disarray due to medial degeneration, vascular wall remodeling, increased wall stiffness, and aortic dilation^[20,21]^. It may indicate a possible increased risk of stroke and CAD rather than hypertrophy^[2]^, and it may be a causative etiology of multiple tiny aneurysms of the cerebral arteries, responsible for pediatric acute ischemic stroke^[22]^. The relationship among SMA, ACTA2 gene, and contractile property in vasculopathies is listed in Figure 1.

Syndromic aneurysms (Marfan syndrome and other connective tissue diseases including Loeys-Dietz syndrome, Ehlers-Danlos syndrome, and familial TAAD) have been proven to have overlapping clinical features and mutated TGF-β genes^[23,24]^ while familial non-syndromic patients are associated with mutations in MYH11, transforming growth factor beta-receptor 1 (TGFβR1), Transforming growth factor beta-receptor 2 (TGFβR2), myosin light chain kinase (MYLK), and ACTA2 genes in spite of incomplete penetrance and/or locus heterogeneity^[24]^. Casual mutations of fibrillin-1, TGFβR1, TGFβR2, ACTA2, MYH11, and SMAD3 genes were detected in TAAD patients^[23,25,26]^.

A mutation in a single ACTA2 gene can cause a variety of vascular diseases^[2]^. Guo et al.^[27]^ reported that mutations in ACTA2 gene are the most common mutations, accounting for 10-15% of familial TAAD mutations. By contrast, only 0.9% of the patients with TAAD had a mutation in the ACTA2 gene according to Lerner-Ellis et al.^[28]^. The prevalence of ACTA2 mutations was 12-16% in familial TAAD^[21,29]^ as opposed to 0% in sporadic cases^[21]^. In patients with known TAAD, non-synonymous missense mutations were identified in 3.85% probands^[30]^. The TAAD family members showed a penetrance as low as 0.48% in heterozygous ACTA2 mutations^[31]^. TAAD was the primary vascular disease in ACTA2-mutation carriers, some of which also had premature CAD, ischemic strokes and multiple vascular diseases, including Moyamoya disease^[2,32,33]^. However, none of the family members without ACTA2 mutation had TAAD, premature CAD or stroke^[2]^. Causative gene mutations for TAAD have been identified, including TGFβR2 at the TAAD2 locus, MYH11 at the 16p locus, and ACTA2 at the TAAD4 locus^[34]^. Causative genes responsible for vasculopathies such as aortic, coronary, peripheral vascular and cerebrovascular disorders were carefully depicted in Table 1 ^[2,20,30,31,34-41]^.

**Table 1:** Causative ACTA2 gene mutations of vasculopathies.

Causative gene mutation	Typical manifestation	Reference
p.Arg64Lys (c.191G>A) (exon 3), p.Arg179Cys (c.535C>T) (exon 6) & p.Lue244Phe (c.732G>T) (exon 7)	TAAD	[23]
p.Arg179His	TAAD	[23]
G304R (in the vicinity of the ATP-binding site)	TAAD, isolated	[20]
p.G152_T205del (c.616+1G>T), p.R212Q & p.R149C	TAAD, familial	[17]
p.Y145C	TAAD, sporadic & young-onset	[17]
c.76G>T; p.Asp26Tyr	TAAD, non-syndromic familial	[19]
R39C (the DNAse-I-binding loop within subdomain 2 of a-smooth muscle actin)	Aortic aneurysm, recurrent	[20]
M49V (the DNAse-I-binding loop within subdomain 2 of a-smooth muscle actin) R118Q & R149C	Aortic dissection	[20]
R118Q & R149C	Aortic disease, coronary artery disease (early onset)	[15]
R258	Aortic disease, patent ductus arteriosus, cerebrovascular disease (very early onset), including Moyamoya disease	[15]
R179H	Aortic & cerebrovascular disease, fixed dilated pupils, hypotonic bladder, gut malrotation, hypoperistalsis & pulmonary hypertension	[15]
R179H	More severe vasculopathy, thoracic aortic aneurysm & brachial artery occlusion	[16,18]
p.R118Q, p.R149C	Coronary artery disease	
p.R179C amino acid substitution (c.535C>T in exon 6)	Primary pulmonary hypertension, persistent ductus arteriosus, extensive cerebral white matter lesions, fixed dilated pupils, intestinal malrotation & hypotonic bladder	[31]
c.772C/T, p.R258C; c.773 G/A, p.R258H; R179H, c.536 G/R (Exon 6)	Moyamoya disease	[33]
p.R258C/H	Strokes	
p.R39H (SD2 domain)	Stroke (before the age of 20)	
R179H substitutions	Cerebral developmental defects (underdeveloped corpus callosum & vermis hypoplasia), vascular fragility & ductus arteriosus rupture	[31]
R179H	Neonatal seizures due to multifocal infarcts, asymmetric motor deficits, global developmental delay, spasticity, congenital bilateral mydriasis & patent ductus arteriosus	[30]
p.R149C, p.R118Q & p.T353N (within the hydrophobic cleft of a-smooth muscle actin)	Premature coronary artery disease	
p.R185Q	Perturb adenosine triphosphatase hydrolysis	
R149C	Skin rash caused by dermal capillary and small artery occlusion referring to as livedo reticularis & with iris flocculi, patent ductus arteriosus & bicuspid aortic valve	

ATP=adenosine triphosphate; TAAD=thoracic aortic aneurysm and dissection.

Heterozygous missense mutations in ACTA2 disrupting Arg179 had persistent ductus arteriosus and congenital mydriasis, variable presentation of pulmonary hypertension, bladder and gastrointestinal disorders, as well as carotid and cerebrovascular abnormalities, including absent basal "Moyamoya"collaterals^[42]^. MYH11 and ACTA2 missense mutations were associated with upregulation of the TGF-β signaling pathway^[29]^. Distinct loci for large pedigrees of familial TAAD were on 5q13-q14, 16p13.12-p13.13, 11q23.3-q24, 3p24-p25, 9q22, 10q22-q24, and 15q24-q26 (MIM 607087, 132900, 607086, 6103080, 608967, 611788, and unassigned), with four being identified as ACTA2 (MIM 102620, 10q23), MYH11 (MIM160745, 16p13), TGFβR1 (MIM 190181, 9q22), and TGFβR2 (MIM 190182, 3p22)^[43]^. Patients presenting with acute ascending or descending aortic dissections have also been found to have ACTA2 mutations^[27]^. Half of the mutation carriers have no aortic disease, while those with aortic aneurysms usually have missense ACTA2 mutations with fewer deletions and splice-site mutations^[19]^. Aortic aneurysms pending to rupture may show various aortic dimensions, in particular, patients with aortic aneurysm with ACTA2 mutations have an aortic dimension of <5.0 cm prior to dissection. Therefore, early surgical treatment is recommended in such patients, even with minimal changes to aortic dimensions^[2]^. Shimojima et al.^[44]^ sequenced the nine exons of ACTA2 in a cohort of 53 patients with Moyamoya disease from 7 familial series without showing any mutations. Similarly, Lee et al.^[45]^ also reported that mutations in human RNF213 were present whereas ACTA2 gene mutations were absent in 36 Taiwanese Moyamoya patients.

### Basic Research for Possible Mechanisms

α-SMA is important for contractile functions of the developing heart at an early embryonic stage. Research on avian epimyocardial cells demonstrated α-SMA mRNAs were mainly detected in conus arteriosus and the ventral aorta before Hamburger-Hamilton stage (HH) 12. In subsequent stages, the α-SMA mRNA was apparently confined to SMCs of the vascular system. The contractive properties of SMCs can be suspended while secretory functions prevail by exertions of mitogens, including platelet-derived growth factor (PDGF) and basic fibroblast growth factor (bFGF), and extracellular matrix components such as collagen, elastin and proteoglycans that contribute to SMC differentiation and proliferation^[3]^. Hence, α-SMA expression inversely correlates with cell proliferation, but directly correlates with contractile efficiency of SMCs^[46]^.

Fibronectin and laminin are expressed typically in the arterial media during fetal and adult life in different forms, and a paradoxical transformation of the proteins from adult to fetal variants takes place during the vascular pathological processes. Vascular SMCs are also implicated in vessel remodeling, in either physiological or pathological conditions, such as pregnancy, exercise, or vascular injury^[47]^. During the remodeling process after vascular injury, the SMCs resume a contractile phenotype, with a cytoplasm dominated by α-actin filaments until the final extent of neointimal formation. The phenotype of cultured rabbit SMCs can be modified by PDGF-BB, resulting in faster growth, but SMCs preincubated with conditioned medium of macrophages plus anti-PDGF antibody did not grow faster. Human arterial and venous SMCs exhibited very different proliferative responses to PDGF isoforms. Proliferation of arterial SMCs was strongly stimulated by PDGF-AA, but venous SMCs showed no proliferative response to PDGF-AA, demonstrating instead a significantly greater proliferative response to PDGFBB than arterial SMCs^[48]^. In cultured rat aortic SMCs, enhanced proliferation, elongated cellular deformity as well as a fluctuation pattern of growth under the exertion of TGF-β_1_ were present^[49]^.

During angiogenesis and vascular remodeling due to restenosis and atherosclerosis, SMCs regulate endothelial cell quiescence and angiogenic responsiveness to cytokines. A series of studies revealed TGF-β stimulates the matrix production of vascular SMCs, including type I collagen. The enhanced α-SMA expression induced by TGF-β_1_ increases the contraction of collagen gel mediated by bovine corneal fibroblasts in a dose-dependent manner. Integrin α_2_β_1_, one of the collagen-binding receptors, binds fibril-forming collagens, collagens I, II, III and X. It joins the process of TGF-β_1_-induced α-SMA production and regulation, capable of generating intracellular tension.

α-SMA disruption results in SMC hyperplasia via certain cellular pathways involving FAK, p53 and PDGF receptor-β, contributing to vascular occlusive disorders in patients with ACTA2 missense mutations. This is supported by the experiments in ACTA2 knockout mice, in which decreased aortic contractility was displayed^[50]^. The structural heterogeneity of genomic DNA encoding the chicken α-SMA gene in 3' untranslated regions is of alternative polyadenylation signals, but with uncertain functional implications. Moreover, actin isoform synthesis after balloon induced endothelial denudation warrants further investigations in order to be employed early in clinical practice.

## CONCLUSION

ACTA2 mutations are associated with structural disruption and functional impairment of contractile proteins, and predispose to a variety of diffuse vascular diseases including TAAD, CAD, ischemic strokes, and Moyamoya disease. Vascular SMCs are also implicated in vascular remodeling in both physiological and pathological conditions. Regulation of differentiation and proliferation of SMCs may control the expression of genes encoding for proteins responsible for the contractile function of the SMCs. In the process of vascular remodeling, contractive properties of SMCs can be suspended while secretory functions prevail by mitogen impacts. Research on α-SMA and ACTA2 mutations is imperative for understanding the pathogenesis and determining the pertinent management strategies of vasculopathies. Further in-depth studies of causative genes of ACTA2 mutations may largely facilitate the diagnosis and treatment of the underlying disorders.

**Table:** 

**Authors' roles & responsibilities**	
SMY	Study conception and design; analysis and/or interpretation of data; manuscript writing, final approval of the manuscript.

## References

[1] Wang J, Zohar R, McCulloch CA (2006). Multiple roles of α-smooth muscle actin in
mechanotransduction. Exp Cell Res.

[2] Guo DC, Papke CL, Tran-Fadulu V, Regalado ES, Avidan N, Johnson RJ (2009). Mutations in smooth muscle alpha-actin (ACTA2) cause coronary
artery disease, stroke, and Moyamoya disease, along with thoracic aortic
disease. Am J Hum Genet.

[3] Milewicz DM, Kwartler CS, Papke CL, Regalado ES, Cao J, Reid AJ (2010). Genetic variants promoting smooth muscle cell proliferation can
result in diffuse and diverse vascular diseases: evidence for a hyperplastic
vasculomyopathy. Genet Med.

[4] Cherng S, Young J, Ma H (2008). Alpha-smooth muscle actin (α-SMA). J Am Sci.

[5] Hinz B, Celetta G, Tomasek JJ, Gabbiani G, Chaponnier C (2001). Alpha-smooth muscle actin expression upregulates fibroblast
contractile activity. Mol Biol Cell.

[6] Suh JH, Yoon JS, Kim HW, Jo KH (2011). Adventitial fibroblast abnormality in thoracic aortic aneurysms
and aortic dissections. Korean J Thorac Cardiovasc Surg.

[7] Schildmeyer LA, Braun R, Taffet G, Debiasi M, Burns AE, Bradley A (2000). Impaired vascular contractility and blood pressure homeostasis in
the smooth muscle alpha-actin null mouse. FASEB J.

[8] Zhang J, Schmidt J, Ryschich E, Schumacher H, Allenberg JR (2003). Increased apoptosis and decreased density of medial smooth muscle
cells in human abdominal aortic aneurysms. Chin Med J (Eng).

[9] Virmani R, Kolodgie FD, Burke AP, Finn AV, Gold HK, Tulenko TN (2005). Atherosclerotic plaque progression and vulnerability to rupture:
angiogenesis as a source of intraplaque hemorrhage. Arterioscler Thromb Vasc Biol.

[10] Tondeleir D, Vandamme D, Vandekerckhove J, Ampe C, Lambrechts A (2009). Actin isoform expression patterns during mammalian development
and in pathology: insights from mouse models. Cell Motil Cytoskeleton.

[11] Wassef M, Baxter BT, Chisholm RL, Dalman RL, Fillinger MF, Heinecke J (2001). Pathogenesis of abdominal aortic aneurysms: a multidisciplinary
research program supported by the National Heart, Lung, and Blood
Institute. J Vasc Surg.

[12] Guo DC, Papke CL, He R, Milewicz DM (2006). Pathogenesis of thoracic and abdominal aortic
aneurysms. Ann N Y Acad Sci.

[13] Hao H, Gabbiani G, Camenzind E, Bacchetta M, Virmani R, Bochaton-Piallat ML (2006). Phenotypic modulation of intima and media smooth muscle cells in
fatal cases of coronary artery lesion. Arterioscler Thromb Vasc Biol.

[14] Bingley JA, Hayward IP, Campbell GR, Campbell JH (2001). Relationship of glycosaminoglycan and matrix changes to vascular
smooth muscle cell phenotype modulation in rabbit arteries after acute
injury. J Vasc Surg.

[15] Toma N, Matsushima S, Murao K, Kawaguchi K, Imanaka-Yoshida K, Yoshida T (2003). Histopathological findings in a human carotid artery after stent
implantation. Case report. J Neurosurg.

[16] Fukui D, Miyagawa S, Soeda J, Tanaka K, Urayama H, Kawasaki S (2003). Overexpression of transforming growth factor ß1 in smooth
muscle cells of human abdominal aortic aneurysm. Eur J Vasc Endovasc Surg.

[17] John Welsh Cardiovascular Diagnostic Laboratory ACTA2 mutation analysis.

[18] Bergeron SE, Wedemeyer EW, Lee R, Wen KK, McKane M, Pierick AR (2011). Allele-especific effects of thoracic aortic aneurysm and
dissection α-smooth muscle actin mutations on actin
function. J Biol Chem.

[19] Wang L, Guo DC, Cao J, Gong L, Kamm KE, Regalado E (2010). Mutations in myosin light chain kinase cause familial aortic
dissections. Am J Hum Genet.

[20] Al-Mohaissen M, Allanson JE, O'Connor MD, Veinot JP, Brandys TM, Maharajh G (2012). Brachial artery occlusion in a young adult with an ACTA2 thoracic
aortic aneurysm. Vasc Med.

[21] Disabella E, Grasso M, Gambarin FI, Narula N, Dore R, Favalli V (2011). Risk of dissection in thoracic aneurysms associated with
mutations of smooth muscle α-actin 2 (ACTA2). Heart.

[22] Amans MR, Stout C, Fox C, Narvid J, Hetts SW, Cooke DL (2013). Cerebral arteriopathy associated with Arg179His ACTA2
mutation. BMJ Case Rep.

[23] Jondeau G, Boileau C (2012). Genetics of thoracic aortic aneurysms. Curr Atheroscler Rep.

[24] Pomianowski P, Elefteriades JA (2013). The genetics and genomics of thoracic aortic
disease. Ann Cardiothorac Surg.

[25] Proost D, Vandeweyer G, Meester JA, Salemink S, Kempers M, Ingram C (2015). Performant mutation identification using targeted next generation
sequencing of fourteen thoracic aortic aneurysm genes. Hum Mutat.

[26] Ware SM, Shikany A, Landis BJ, James JF, Hinton RB (2014). Twins with progressive thoracic aortic aneurysm, recurrent
dissection and ACTA2 mutation. Pediatrics.

[27] Guo DC, Pannu H, Tran-Fadulu V, Papke CL, Yu RK, Avidan N (2007). Mutations in smooth muscle α-actin (ACTA2) lead to
thoracic aortic aneurysms and dissections. Nat Genet.

[28] Lerner-Ellis JP, Aldubayan SH, Hernandez AL, Kelly MA, Stuenkel AJ, Walsh J (2014). The spectrum of FBN1, TGFßR1, TGFßR2 and ACTA2
variants in 594 individuals with suspected Marfan Syndrome, Loeys-Dietz
Syndrome or Thoracic Aortic Aneurysms and Dissections (TAAD). Mol Genet Metab.

[29] Renard M, Callewaert B, Baetens M, Campens L, MacDermot K, Fryns JP (2013). Novel MYH11 and ACTA2 mutations reveal a role for enhanced
TGFß signaling in FTAAD. Int J Cardiol.

[30] Arno G, Aragon-Martin JA, Harris S Mutations in ACTA2 in a British cohort of TAAD patients.

[31] Milewicz DM, Guo DC, Tran-Fadulu V, Lafont AL, Papke CL, Inamoto S (2008). Genetic basis of thoracic aortic aneurysms and dissections: focus
on smooth muscle cell contractile dysfunction. Annu Rev Genomics Hum Genet.

[32] Hoffjan S (2012). Genetic dissection of Marfan syndrome and related connective
tissue disorders: an update 2012. Mol Syndromol.

[33] Katerina L Byanova. Effects of the ACTA2 R258C mutation on vascular smooth muscle
cell phenotype and properties. UT GSBS Dissertations and Theses.

[34] Al-Mohaissen M, Allanson JE, O'Connor MD, Veinot JP, Brandys TM, Maharajh G (2012). Brachial artery occlusion in a young adult with an ACTA2 thoracic
aortic aneurysm. Vasc Med.

[35] Milewicz DM, Østergaard JR, Ala-Kokko LM, Khan N, Grange DK, Mendoza-Londono R (2010). De novo ACTA2 mutation causes a novel syndrome of multisystemic
smooth muscle dysfunction. Am J Med Genet A.

[36] Morisaki H, Akutsu K, Ogino H, Kondo N, Yamanaka I, Tsutsumi Y (2009). Mutation of ACTA2 gene as an important cause of familial and
nonfamilial nonsyndromatic thoracic aortic aneurysm and/or dissection
(TAAD). Hum Mutat.

[37] Yoo EH, Choi SH, Jang SY, Suh YL, Lee I, Song JK (2010). Clinical, pathological, and genetic analysis of a Korean family
with thoracic aortic aneurysms and dissections carrying a novel Asp26Tyr
mutation. Ann Clin Lab Sci.

[38] Hoffjan S, Waldmüller S, Blankenfeldt W, Kötting J, Gehle P, Binner P (2011). Three novel mutations in the ACTA2 gene in German patients with
thoracic aortic aneurysms and dissections. Eur J Hum Genet.

[39] Moosa AN, Traboulsi EI, Reid J, Prieto L, Moran R, Friedman NR (2013). Neonatal stroke and progressive leukoencephalopathy in a child
with an ACTA2 mutation. J Child Neurol.

[40] Meuwissen ME, Lequin MH, Bindels-de Heus K, Bruggenwirth HT, Knapen MF, Dalinghaus M (2013). ACTA2 mutation with childhood cardiovascular, autonomic and brain
anomalies and severe outcome. Am J Med Genet A.

[41] Roder C, Peters V, Kasuya H, Nishizawa T, Wakita S, Berg D (2011). Analysis of ACTA2 in European Moyamoya disease
patients. Eur J Paediatr Neurol.

[42] Munot P, Saunders DE, Milewicz DM, Regalado ES, Ostergaard JR, Braun KP (2012). A novel distinctive cerebrovascular phenotype is associated with
heterozygous Arg179 ACTA2 mutations. Brain.

[43] Prakash SK, LeMaire SA, Guo DC, Russell L, Regalado ES, Golabbakhsh H (2010). Rare copy number variants disrupt genes regulating vascular
smooth muscle cell adhesion and contractility in sporadic thoracic aortic
aneurysms and dissections. Am J Hum Genet.

[44] Shimojima K, Yamamoto T (2009). ACTA2 is not a major disease-causing gene for moyamoya
disease. J Hum Genet.

[45] Lee MJ, Chen YF, Fan PC, Wang KC, Wang K, Wang J (2015). Mutation genotypes of RNF213 gene from moyamoya patients in
Taiwan. J Neurol Sci.

[46] Hinz B, Celetta G, Tomasek JJ, Gabbiani G, Chaponnier C (2001). Alpha-smooth muscle actin expression upregulates fibroblast
contractile activity. Mol Biol Cell.

[47] Owens GK, Kumar MS, Wamhoff BR (2004). Molecular regulation of vascular smooth muscle cell
differentiation in development and disease. Physiol Rev.

[48] Li L, Blumenthal DK, Terry CM, He Y, Carlson ML, Cheung AK (2011). PDGF-induced proliferation in human arterial and venous smooth
muscle cells: molecular basis for differential effects of PDGF
isoforms. J Cell Biochem.

[49] Orlandi A, Ropraz P, Gabbiani G (1994). Proliferative activity and a-smooth muscle actin expression in
cultured rat aortic smooth muscle cells are differently modulated by
transforming growth factor-ß1 and heparin. Exp Cell Res.

[50] Schildmeyer LA, Braun R, Taffet G, Debiasi M, Burns AE, Bradley A (2000). Impaired vascular contractility and blood pressure homeostasis in
the smooth muscle α-actin null mouse. FASEB J.

